# Modern trends in breeding and genetic improvement of sunflower varieties and hybrids at VNIIMK

**DOI:** 10.18699/VJ21.042

**Published:** 2021-07

**Authors:** V.M. Lukomets, M.V. Trunovа, Ya.N. Demurin

**Affiliations:** V.S. Pustovoit All-Russian Research Institute of Oil Crops, Krasnodar, Russia; V.S. Pustovoit All-Russian Research Institute of Oil Crops, Krasnodar, Russia; V.S. Pustovoit All-Russian Research Institute of Oil Crops, Krasnodar, Russia

**Keywords:** sunflower, breeding, variety, hybrid, early maturity, resistance to pathogens, large-fruited, herbicide resistance, ornamental, подсолнечник, селекция, сорт, гибрид, скороспелость, устойчивость к патогенам, крупноплодность, гербицидоустойчивость, декоративность

## Abstract

Modern sunflower breeding is significantly diversified due to the different needs of agricultural production. The breeding of sunflower varieties and hybrids is carried out at V.S. Pustovoit All-Russian Research Institute of Oil Crops (VNIIMK) in all areas in demand on the market and is based on fundamental biological research. In the field of breeding for faster maturing, the following commercial cultivars were obtained: very early maturing, cv. Skormas and the three-way hybrid Achilles, early maturing cvs. Varyag and Uspekh, medium maturing cvs. Amelie, Aris and Aurus. Within the framework of breeding for immunity, eight hybrids and one variety have been produced. So at the Don experimental station (Rostov region), productive hybrids were bred, resistant to the virulent broomrape of the G race due to the presence of the Or7 gene: ‘Gorstar’, ‘Gorfild’, ‘Grant’, ‘Status’, ‘Fogor’ and the three-way hybrid Nika. On the central experimental base, the following were obtained: the mid-early hybrid Typhoon and the earlymaturing variety Platonych with resistance to common races of downy mildew and a high oil content of achenes (up to 53 %) as well as the mid-early hybrid Tayzar, which is simultaneously resistant to virulent races of broomrape and to the causative agent of downy mildew. The early maturing large-fruited sunflower variety Belochka was included in the “Russian State Register of Selection Achievements…”, and the large-fruited varieties Karavan, Konditer and Kalibr are currently undergoing state tests. The breeding use of germplasm with genes for herbicide resistance was accompanied by their extensive genetic study. A practical recommendation for all three alleles of the ALS gene (Imr, CLHA-Plus, Sur) was the need to create homozygous hybrids for their reliable use in appropriate production systems. For Clearfield technology, the hybrids Imidzh, Arimi and Immi have been developed; for Clearfield Plus, the hybrid Klip; and for Express Sun (or SUMO), the hybrid Surus. Klip and Surus are mid-oleic. All newly developed fertile ornamental sunflower varieties – Aurelia, Fizalia, Zhemchuzhny, Rumyanets, Agat and Mazhor – were transferred for practical use to a sterile CMS RIG basis. Thus, new achievements have been attained across the entire spectrum of modern trends in sunflower breeding.

## Abstract (ru)

Современная селекция подсолнечника сильно диверсифицирована различными потребностями сельскохозяйственного производства. Во Всероссийском научно-исследовательском институте масличных культур (ВНИИМК) селекция сортов и гибридов подсолнечника ведется по всем востребованным на рынке направлениям и основывается на фундаментальных биологических исследованиях. В направлении селекции на сокращение вегетационного периода растений получены: скороспелые сорт Скормас и трехлинейный гибрид Ахиллес, раннеспелые сорта Варяг и Успех, среднеспелые гибриды Амели, Арис и Аурус. При селекции на устойчивость к болезням созданы восемь гибридов и один сорт. Так, на Донской опытной станции выведены продуктивные гибриды, устойчивые к вирулентной заразихе расы G за счет наличия гена Or7: Горстар, Горфилд, Грант, Статус, Фогор и трехлинейный гибрид Ника. На центральной экспериментальной базе получены среднеранний гибрид Тайфун и раннеспелый сорт Платоныч с устойчивостью к распространенным расам ложной мучнистой росы и с высокой масличностью семянок (до 53 %), а также среднеранний гибрид подсолнечника Тайзар, который обладает устойчивостью к вирулентным расам заразихи и к возбудителю ложной мучнистой росы. В «Государственный реестр селекционных достижений…» внесенскороспелый крупноплодный сорт подсолнечника Белочка; проходят госсортоиспытание крупноплодные сорта Караван, Кондитер и Калибр. Результатом генетического изучения генов гербицидоустойчивости стала практическая рекомендация для применения в селекции подсолнечника трех генов устойчивости к ингибиторам ацетолактатсинтетазы (Imr, CLHA-Plus, Sur), которая заключается в необходимости создания гомозиготных гибридов для их надежного использования в соответствующих производственных системах. Для технологий Clearfield получены гибриды Имидж, Арими и Имми, для Clearfield Plus – гибрид Клип, а для Express Sun (или SUMO) – гибрид Сурус. Гибриды Клип и Сурус являются среднеолеиновыми по качеству масла. Все созданные фертильные декоративные сорта подсолнечника Аурелия, Физалия, Жемчужный, Агат, Мажор и гибрид Румянец переведены для практического использования на стерильную основу ЦМС RIG. Таким образом, по всему спектру современных направлений селекции подсолнечника во ВНИИМК имеются новые достижения

## Introduction

Throughout the more than a century-old history of sunflower (Helianthus annuus L.) breeding in Russia, which began at V.S. Pustovoit All-Russian Research Institute of Oil Crops (VNIIMK) in 1912, several developments of the Institute’s scientists have obtained a clear global priority. First of all, it is the academician V.S. Pustovoit’s development of a practically new field crop of oil sunflower with oil content in achenes of up to 50 %. After that, there was a focused effort to change the fatty acid composition of sunflower oil, which led to the development of the world’s first high-oleic variety Pervenets (Škorić et al., 2012). These two most significant breeding achievements formed the basis of the modern gene pool of oil sunflower and breeding trends in the world and significantly influenced the work of the agro-industrial complex of many countries.

Currently, at VNIIMK, both varieties-populations and the interline hybrids of sunflower are bred. For 2020, 41 sunflower varieties of VNIIMK’s breeding have been included into the “State Register of Selection Achievements…” of the Russian Federation, which is 41 % of the total number of varieties permitted for cultivation in the country (State Register…, 2020). The sunflower hybrids (simple and three-way) are represented by 58 genotypes, which is 9 % of their total number. A total of 99 sunflower varieties and hybrids of VNIIMK’s breeding occupy 14 % of their total number in the “State Register…”. Moreover, among 16 varieties of ornamental sunflower included in the “State Register…” six ones are of VNIIMK’s breeding, which is 38 %.

The modern sunflower breeding is largely diversified by various challenges of agricultural production and directions of crop usage (Škorić et al., 2012). On the other hand, the success of breeding at VNIIMK has always been based on fundamental agrobiological research, including efficient methods for evaluation of traits and selection of desired genotypes. This article presents the main recent results of this work (see the Table). 

**Table 1.**
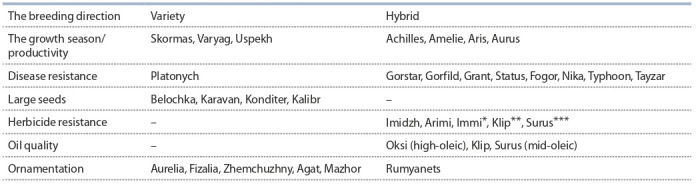
The latest achievements of VNIIMK in sunflower breeding * For Clearfield cultivation technology; ** for Clearfield Plus cultivation technology; *** for SUMO cultivation technology

## Breeding for decreasing the length of the growth season

Decreasing the period from seedling emergence to physiological maturity of sunflower, largely controlled genetically, allows expanding the crop acreage and carrying out replanting or resowing crops in one year. The main problem of using such cultivation technology lies in minimizing the yield decrease and not in the absence of hereditary variability in the length of the growth season. The period from physiological to harvesting (technical) maturity mainly depends on environmental factors, including air temperature, precipitation, and desiccation, and can vary from 14 to 20 days. 

At VNIIMK, Krasnodar, the early-maturing variety-population Skormas was developed from the oil variety SUR by the classical breeding scheme, specifically by the method of multiple individual selection with an evaluation of progeny and subsequent pollination of the best families in terms of a complex of traits. The period from seedling emergence to physiological maturity of the variety Skormas is 79 days with a productivity of 2.96 t/ha and oil content of achenes of 50 % (Detsyna, Illarionova, 2018).

At the Siberian experimental station of VNIIMK (Isilkul, Omsk region), two early-maturing sunflower varieties for cultivation in the extreme conditions of Western Siberia were developed. One of them, the variety Varyag, was developed by the method of multiple self-pollination of plants of the variety Skorospely-87 and the use of individual selection, followed by pollination within the best families during free flowering. The period from seedling emergence to physiological maturity is 94 days with a productivity of 3.15 t/ha and oil content of achenes of 52 % (Puzikov, Suvorova, 2018). Another variety, Uspekh, was developed using individual selection from hybrids obtained by crossing the early-maturing varieties Rodnik and Ermak, followed by cross-pollination of the best families during free flowering. The period from seedling emergence to physiological maturity is 98 days with a productivity of 3.47 t/ha and oil content of achenes of 55 %. The variety Uspekh has one of the highest oil contents among the varieties of its region

In addition, at VNIIMK, an early-maturing three-way hybrid Achilles was developed, which showed the growth season of 76 days with a productivity of 3.41 t/ha and oil content of achenes of 49 % in trial conditions of Krasnodar.

The sunflower hybrids of mid-maturing group are characterized by the highest productivity. At the Armavirskaya experimental station, the breeding in this direction led to the development of highly productive hybrids Amelie, Aris and Aurus. 

## Breeding for disease resistance

Numerous sunflower diseases can significantly decrease the productivity and seed quality. The accelerating racial evolution of both the flower parasite of sunflower broomrape Orobanche cumana Wallr. (Khatnyanskij, 2020) and the obligate pathogen of downy mildew Plasmopara halstedii (Farl.) Berl. et de Toni makes breeding for resistance to them a constant process.

We revealed an incomplete dominance of the resistance gene Or7 to the new virulent broomrape race G during the hybridological analysis carried out at VNIIMK (Guchetl et al., 2019). Moreover, we continue the search for molecular genetic markers loci that control broomrape resistance. Similar work is being carried out with genes that determine resistance to the downy mildew pathogen (Ramazanova et al., 2020).

In breeding for immunity, eight hybrids and one sunflower variety have been developed at VNIIMK in recent years. For example, at the Don experimental station, the following productive hybrids resistant to broomrape race G due to the presence of the Or7 gene were developed: Gorstar (Gorbachenko et al., 2018), Gorfild, Grant, Status, Fogor (maximum broomrape resistance) and three-way hybrid Nika (Gorbachenko et al., 2020).

At VNIIMK, Krasnodar, were developed the mid-early maturing hybrid Typhoon with resistance to common races of downy mildew and a high oil content of seeds – up to 53 %, and the early-maturing variety Platonych with similar oil content and resistance to downy mildew (Detsyna, Illarionova, 2019).

**Fig. 1.**
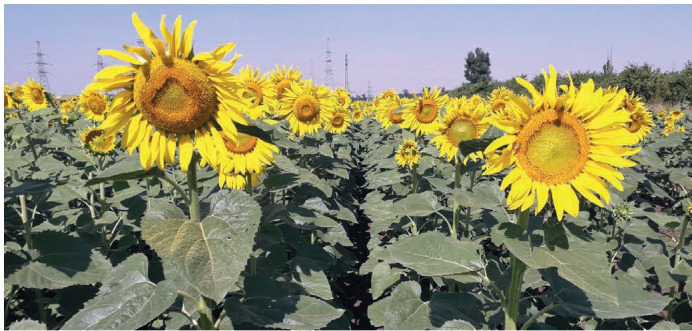
Sunflower hybrid Tayzar at the flowering stage. VNIIMK’s field (Krasnodar), photo by the authors.

The latest breeding achievement is the mid-early maturing productive hybrid Tayzar (Fig. 1), which has been submitted for State variety trial in 2021; it is simultaneously resistant to the virulent broomrape race G and five races of the downy mildew pathogen – 330, 710, 730, 334, and 734 (Demurin et al., 2020c).

## Breeding of large-seeded varieties

The feature of Russian varieties of confectionery sunflower, such as SPK, Dzhinn, Lakomka, and Oreshek, is their intermediate place in terms of achenes between edible and oil forms. Exactly this type of achene is in demand in Russia and the CIS countries. The study of the inheritance of the trait “seed size”, estimated as a thousand-seed weight, showed polygenic control and a strong dependence of its expression on the applied cultivation technology (plant population)

In 2018, the new early-maturing large-seeded sunflower variety Belochka developed at VNIIMK was included into the “State Register of Selection Achievements…” (Detsyna et al., 2018). It was developed using the method of multiple individual selection with an evaluation of the progeny and pollination within the best families in terms of economic valuable traits, including broomrape resistance. In terms of productivity, the variety exceeded the standard variety Oreshek by 0.16 t/ha, with an average decrease of the growth season by three days. The variety is characterized by uniformity of flowering and maturing dates, with a thousand-seed weight of about 100 g with a plant population of 40 thousand pcs/ha

Currently, new, more productive, large-seeded varieties Karavan, Konditer, and Kalibr are undergoing the State variety trial.

## Breeding for herbicide resistance

Currently, for sunflower cultivation, three production systems of growing “hybrid–herbicide” were developed and are widely used, both in our country and abroad: Clearfield, Clearfield Plus, and Express Sun (or SUMO), based on the use of the Imr, CLHA-Plus, and Sur genes, respectively (Škorić et al., 2012). 

At VNIIMK, the use of obtained sources with herbicide resistance genes in breeding was followed by their genetic study. In particular, we received important data on the dominance type of resistance to the selective herbicide tribenuron-methyl from the sulfonylurea class at various doses of the active ingredient (Demurin et al., 2016). The main practical recommendation when using the Imr, CLHA-Plus and Sur genes is the necessity to develop homozygous parent lines and hybrids for their reliable use in the mentioned production systems. 

Imidzh and Arimi, the first Russian imidazolinone-resistant hybrids of the VNIIMK breeding suitable for cultivation with Clearfield technology, have been included into the “State Register of Selection Achievements…” since 2014. The Immi hybrid, also homozygous by the Imr gene and adapted for this cultivation technology, is currently undergoing the State variety trial.

A simple interline sunflower hybrid Klip was developed as part of a breeding and genetic program for the development of herbicide-resistant plants for growing by the Clearfield Plus production system. This hybrid, like its parental forms, is homozygous by the CLHA-Plus imidazolinone resistance gene. The hybrid belongs to the mid-early group of maturity, has high seed productivity, is resistant to racesA–E of broomrape and to race 330 of downy mildew, and tolerant to Phomopsis blight caused by Phomopsis helianthi Munt. The period from seedling emergence to harvesting maturity is 115 days, the oil content of seeds is 50 %, the huskness is 21 % (Demurin et al., 2020a). The hybrid Klip is undergoing the State variety trial.

For the Express Sun production system was developed and is also undergoing the State variety trial a simple interline sunflower hybrid Surus, which is highly resistant to the tribenuron-methyl herbicide. Both parental forms, as well as the hybrid, are homozygous by the Sur gene. The hybrid Surus belongs to the mid-maturing group, has high productivity, is resistant to broomrape (racesA–E) and downy mildew (race 330), and tolerant to Phomopsis blight. The period from seedling emergence to harvesting maturity is 120 days, the oil content of seeds is 50 %, the huskness is 22 % (Demurin et al., 2020b).

## Breeding for oil quality

The trait of high oleic acid content keeps ranking high in the breeding programs of companies in most countries. The herbicide-resistant hybrids Klip and Surus have a medium oleic segregation type of oil in the commercial seeds of F2 hybrids. Earlier, in 2014, we developed a high oleic hybrid Oksi with a modified composition of tocopherols (vitamin E). Amolecular marker was validated to control the genetic purity of lines with a high oleic content mutation Ol (Guchetl, 2020). 

## Breeding for ornamentality

**Fig. 2.**
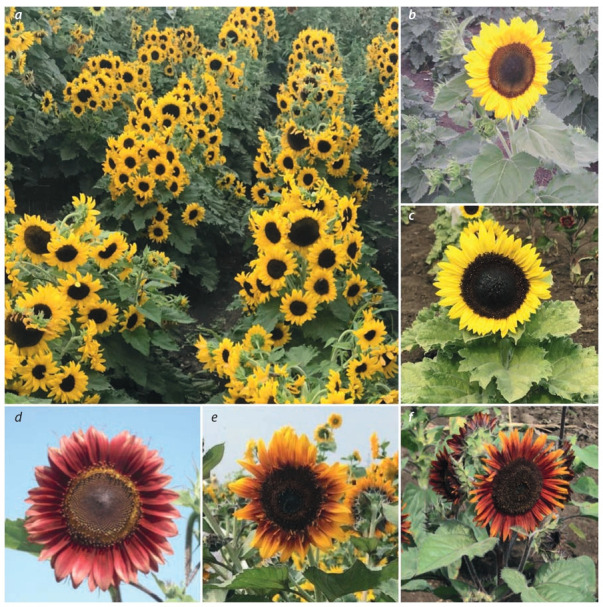
Ornamental varieties of VNIIMK breeding: а, Fizalia; b, Aurelia; с, Zhemchuzhny; d, Rumyanets; e, Mazhor; f, Agat Photo by the authors.

The development of ornamental forms of sunflower at VNIIMK initially led to the breeding of two varieties with an ornamental phenotype in 2016 (Fig. 2). The variety Aurelia was received during the study of the genetic collection by crossing the dwarf sample I5/303 and the ornamental variety, followed by self-pollination and individual selection based on morphotype traits. The main ornamental characteristics of the variety Aurelia (see Fig. 2, b) are low height, compact pyramidic habitus, large number of inflorescences, common branching of the stem, location of the central head above the lateral inflorescences, and long flowering period. The main ornamental characteristics of the variety Fizalia (see Fig. 2, a) are low height, compact cylindric habitus, large number of inflorescences, apical branching of the stem, location of the central head on the same level with the lateral inflorescences, and long flowering period. 

In 2017, the ornamental variety Zhemchuzhny was submitted to the State Commission of the Russian Federation for Selection Achievements Test and Protection. It was received by crossing a dwarf sample LD4 and the line VIR721. Its main ornamental characteristics (see Fig. 2, c) are light yellow color of ligulate florets, light-green leaf with blue-grey tint, low height, compact single-head habitus (Peretyagina et al., 2018). 

The hybrid of ornamental sunflower Rumyanets was received by crossing the parental lines LD110 and LD120. The main ornamental characteristics of the hybrid Rumyanets (see Fig. 2, d ) are common branching of the stem, large number of inflorescences, crimson color of ligulate florets, long flowering period. The hybrid belongs to the late-maturing group. 

In 2019, the ornamental variety Agat was submitted to the State Commission of the Russian Federation for Selection Achievements Test and Protection. It was received by crossing a dwarf sample LD4 and the line LD11. The main ornamental characteristics of the hybrid Agat (see Fig. 2, f  ) are active accumulation of anthocyanins in the mesophyll of young leaves, stems, and husk leaves; purple color of ligualte and tubular florets; large, bladder-like leaves creviced along the edges; low height; compact habitus; long growth season.

The ornamental variety Mazhor was received by crossing the ornamental variety and the line LD11, followed by selfpollination and individual selection based on morphotype traits. The main ornamental characteristics of the variety Mazhor (see Fig. 2, e) are yellow-red color of ligulate and tubular florets; weakly pleiopetalous inflorescence of the head; heavy habitus; large number of inflorescences; long growth season.

All fertile ornamental sunflower varieties have been transferred for practical use to a non-restorable sterile base CMS RIG to protect the copyright of breeders and eliminate the factor of pollen allergenic capacity, since the plants are used both for cutting for bouquets in a confined space, as well as in parks, gardens, and individual land properties. 

## Conclusion

Breeding of sunflower varieties and hybrids for all directions that are in demand on the market for this crop is being conducted at VNIIMK. The breeding process is based on fundamental agrobiological research. The genetic collection of this oil and ornamental crop collected and maintained at VNIIMK provides with the necessary sources and donors of economically valuable traits the breeding throughout the Russian Federation and several CIS countries. Further prospects for sunflower breeding in Russia and in the world will probably be focused on the development of competitive advantages of this crop in comparison with other agricultural plants in terms of profitable production of raw materials for the food industry, as well as for fodder, technical, and ornamental uses.

## Conflict of interest

The authors declare no conflict of interest.
